# Circ-HIPK3 Strengthens the Effects of Adrenaline in Heart Failure by MiR-17-3p - ADCY6 Axis

**DOI:** 10.7150/ijbs.36149

**Published:** 2019-09-07

**Authors:** Yunfei Deng, Jun Wang, Guojin Xie, Xiaochen Zeng, Hongli Li

**Affiliations:** 1 Department of Cardiology, Shanghai General Hospital, School of Medicine, Shanghai Jiao Tong University, Shanghai, China.; 2 Department of Urology, Children's Hospital of Nanjing Medical University, Nanjing, China; 3 Department of Clinical Laboratory, Children's Hospital of Nanjing Medical University, Nanjing, China

**Keywords:** circ-HIPK3, heart failure, miR-17-3p, ADCY6, Ca^2+^

## Abstract

Overactivation of β-adrenergic receptor (β-AR) can improve cardiac function temporarily but promotes the development and mortality of heart failure (HF) in the long run. CircRNA, a member of noncoding RNAs, can tolerate digestion of exonuclease and be a chronic stimulator to cell. But the relationship of circRNA with HF remains a puzzle and needs to be explored. Here, we found that circ-HIPK3 affected the concentration of Ca^2+^ in cytoplasm by miR-17-3p through ADCY6 (Adenylate cyclase type 6). The increase of ADCY6 caused by circ-HIPK3 was ameliorated by miR-17-3p overexpression and vice versa, implicating the existence of circ-HIPK3 - miR-17-3p - ADCY6 axis. And further assays showed that the level of circ-HIPK3 in heart was upregulated by adrenaline via transcription factor CREB1 (cAMP responsive element-binding protein 1). Experiments *in vivo* showed downregulation of circ-HIPK3 can alleviate fibrosis and maintain cardiac function post MI in mice. In conclusion, the increased circ-HIPK3 can be a helper for adrenaline but was harmful for heart in the long run and might be an ideal therapeutic target of HF.

## Introduction

As one of the main death causes worldwide, Heart failure (HF) is a clinical syndrome characterized by typical symptoms (breathless, ankle swelling and fatigue) without effective treatments to cure or slow the exacerbating process.^1^ High resting heart rate (≥ 70 bpm) was common in HF patients because of the continuous stimulation of catecholamine. Activation of β-adrenergic receptor (β-AR) could improve cardiac function via canonical cAMP-PKA-Ca^2+^ pathway but also increase mortality in the long run. As an effective treatment of HF, β-blockers can reduce hospital admissions and mortality rate significantly by blocking the activation of β1-AR.^2^

Ca^2+^, a mediator of systole and diastole of heart, is released or absorbed by sarcoplasmic reticulum (SR) via the ryanodine receptor 2 (RyR2) or sarcoplasmic/ endoplasmic reticulum Ca^2+^ ATPase 2a (SERCA2a) respectively. Previous researches indicated that reduced SR Ca^2+^ load and Ca^2+^ transient amplitude were responsible for the decreased contractility and relaxation of HF.^3^ And aberrant density of Ca^2+^ in cytoplasm can accelerate the remodeling of hearts by induction of atrial fibrillation (AF).^4^ Impaired release or reuptake ability of ER caused by chronic activation of β-AR through PKA phosphorylation provides a mechanism to explain the therapeutic efficacy of β-blockers in HF.^5^ Novel therapeutics of HF focused on both RyR2 and SERCA2a are undergoing clinical testing.

CircRNA, a new member of non-coding RNA, are mainly produced by back-splicing from exons with a covalent bond. It can tolerate the digestion of exonuclease for lacking of 5' cap and 3' poly (A), suggesting that circRNA are much more stable than linear RNA, which mainly locate at cytoplasm and exert its function by acting as a sponge of miRNAs.^6^, ^7^ Most of circRNAs are made of exons, implying that they might have a translational potential. Two reported mechanisms - IRES (Internal Ribosomal Entry Sites) and circRNAs m6A - can initiate the translation of circRNAs.^8^, ^9^ Yibing Yang *et al*. found that a polypeptide translated by a circRNA from gene FBXW7 has potential prognostic implications in brain cancer recently.^10^ Furthermore, a circRNA in blood from a fusion gene could be a novel biomarker for non-small cell lung cancer.^11^ Apart from circRNA, ciRNAs (circular intron RNAs) or EIciRNAs (exon-intron RNAs) mainly lie in nucleus and affect the expression of parental genes by acting as cis-acting elements.^12^, ^13^ Increasing evidences showed that aberrant expression of circRNA are related to various cardiovascular diseases intensively.^14^, ^15^ HIPK3 (Homeodomain-interacting protein kinase 3), one of Serine/threonine-protein kinases, have a top expression level in heart, which was associated with transcription regulation, apoptosis and steroidogenic gene expression.^16^-^18^ Circ-HIPK3 derived from the second exon of HIPK3 was related to gallbladder cancer, osteosarcoma or lens epithelial cells.^19^-^21^ But the relationship between circ-HIPK3 and heart still remains unclear.

In our study, we found that circ-HIPK3 can be upregulated significantly in heart post myocardial infarction (MI) and affected the Ca^2+^ in cytoplasm via circ-HIPK3 - miR-17-3p - ADCY6 axis. Its reduction in heart *in vivo* can maintain the cardiac function post MI and might be a promising therapeutic target of HF in the future.

## Materials and Methods

### Materials

Anti-RyR2, anti-PLN, anti-p-PLN, anti-p-RyR2, anti-His were purchased from Abcam (Britain). Adrenaline and carbamylcholine were from Sangon Biotech (1: 40000, China). Fluo-3/AM was from Beyotime Biotechnology (China). TRIzol reagent was from Invitrogen (USA); a SYBR RT-PCR Kit and DNA PCR kit were from Takara Bio Inc. (Japan); RNase R was from Epicenter (USA); primers, mimic, and siRNA were designed and synthesized by Sangon Biotech (China).

### Animal Models (HF post MI (myocardial infarction))

24 male mice (C57BL/6) were divided into 4 groups randomly including normal group (without surgery), control group (without ligation), NC (negative control) group and experiment group. Briefly, the mice were anesthetized with intraperitoneal sodium pentobarbital (50 mg/kg), underwent thoracotomy and pericardiotomy, and then the left anterior descending (LAD) coronary artery was ligated at its origin with a 6-0 prolene suture after the heart being squeezed out. Adeno-associated virus (AAV) is an efficient and safe vector for *in vivo* gene transfer experiments, and serotype 9 is significantly cardiotropic and has been widely used. To investigate whether circ-HIPK3 is sufficient to improve cardiac function *in vivo*, we injected the mice in the experiment group with the AAV9-shRNA targeting circ-HIPK3 (Obio Technology, Shanghai, China) via tail vein. We injected the mice in the NC group with AAV9-NC. After 6 weeks, all the mice underwent echocardiographic evaluations to assess their cardiac function. The mice were then sacrificed by cervical dislocation and their hearts were used in following experiments. All animal experimental protocols were approved by the Animal Care and Use Committee, Research Institute of Medicine, Shanghai Jiao Tong University, and all animal experiments were conducted in accordance with the guidelines of the National Institutes of Health, 8th edition, 2011. All efforts were made to minimize animal suffering.

### RNA extraction, RNase R Digestion and quality control

Total RNA was extracted from samples using a homogenizer and Trizol regent. Subsequently, the quantification and quality of purified RNA were assessed using a NanoDrop ND-1000 (NanoDrop Technologies; Thermo Fisher Scientific, Inc.). RNase R treatment was used to digest the ribosomal RNAs and linear RNAs (30min 37°C). The integrity of RNA was assessed by electrophoresis on a denaturing 1.5% agarose gel.

### Quantitative real-time polymerase chain reaction (qRT-PCR) analysis and semi- Quantitative PCR analysis

Total RNAs isolated from tissues and cell were reverse transcribed into cDNA for qRT-PCR analysis. Quantitative RT-PCR was performed in ABI QuantStudio™ 6 Flex Real-time PCR systems according to the manufacturer's instructions. A relative expression of 2^(—ΔΔCT)^ compared to the lowest value was used to analyze the gene expression level. Agarose gel electrophoresis was used to analyze the result of semi-Quantitative PCR. The following primers were used for the experiment: circ-HIPK3, forward, 5'- ATCCTGTTCGGCAGCCTTAC-3' and reverse, 5'-TTTCTTCACACTACAAA AGGCAC-3' mice miR-17-3p primer for RT, CTCAACTGGTGTCGTGGAGTCGGCAATTCAGTTGAGCTACAAGT; forward, 5'-CTCAACTGGTGTCGTGGA -3' and reverse, 5'-ACTTGTAGCTCAACT-3'; mice U6, primer for RT, CTCAACTGGTGTCGTGGAGTCGG CAATTCAGTTGAGAAAATATG; forward, 5'- CAA ATTCGTGAAGCGTT -3' and reverse, 5' -TGGTGTCGTGGAGTCG-3'; mice β-actin, forward, 5'-CGACCACACACAGAAGGAGAT-3' and reverse, 5'-GCCGATTCACACCGAGTA-3'; mice PLN, forward, 5'- TTTACAAGATCCAGCCGATGAT-3' and reverse, 5'- CTGCTGATCTGCATCATTGTGA-3'; mice ADCY6, forward, 5'-CCAAACCTGGCCTCCTAAGT-3' and reverse, 5'-CCATCCCTTAGCACAGGAAA-3'; mice RyR2, forward, 5'-TTGTTTCCTTTTTAGAAAATGACTTC-3' and reverse, 5'- GGGTGTGCAGATGTACATGC-3'; mice SERCA2a, forward, 5'- GGTTTTCAGTGGCTGAGGAA-3' and reverse, 5'- TCCTGAGAATCACTGCTCCC-3'. The experiment was repeated 3 times.

### Annotation for circRNA/miRNA/mRNA interactions

In our work, circRNA/miRNA/mRNA interactions were analyzed with Arraystar's home-made miRNA target prediction software based on miRanda (microrna.org/microrna/ home.do) and TargetScan(targetscan.org/vert_71/).^22^ We set the match score higher than 150 and the minimum free energy less than -25 to improve the reliability of our prediction. MiRNAs and mRNAs coexisted in both predicted results were chosen for further study.

### MiRNA, plasmid transfection and RNA interference

To achieve the gain or loss of circ-HIPK3, miR-17-3p and ADCY6, we transfected pGV486- circ-HIPK3 (2ug), si- circ-HIPK3 (50nM), miR-17-3p mimics (50 nM), miR-17-3p inhibitors (100 nM), pCMV-ADCY6 (2ug) and siRNA-ADCY6 (50 nM) (RiboBio, Guangzhou, China), respectively, into NMCMs using Lipofectamine 3000 (Invitrogen, USA,California), according to the manufacturer's instructions. Control cells were transfected with the appropriate negative controls. The sequences of the oligonucleotides were as follows: miR-17-3p mimic: 5'-ACUGCAGUGAGGGCACUUGUAG-3'; miR-17-3p inhibitor: 5'-CUACAAGUGCCCUCACUGCAGU -3'; si-ADCY6: 5'-TCTGCCCGGGCCTCGGGCA-3'; si- circ-HIPK3: 5'- AGGCCATACCTGTAGTAGC-3'.

### Gene Ontology (GO) analysis and Kyoto Encyclopedia of Genes and Genomes (KEGG) pathway analysis

The GO analysis (geneontology.org) is to construct meaningful annotation of gene products in three domains including biological process (BP), cellular components (CC) and molecular function (MF). KEGG (genome.jp/kegg/) was to predict the molecular interactions and reaction networks of the differentially expressed genes. The enrichment score (-log10 (P-value)) represents the significance of GO term enrichment or pathway enrichment among genes which produces differentially expressed circRNA or the targets of the circRNA.

### TTC staining and histological analysis

Hearts were excised and sectioned transversely into six sections, and then incubated in 2% triphenyltetrazolium chloride (TTC, Sigma-Aldrich, USA) for 10 min at 37 ℃, followed by 10% neutral-buffered formaldehyde for 24 h. Sections were weighed and photographed using a Leica microscope, then analyzed using Image J (National Institutes of Health). Viable myocardium stained red, and the infarcted areas appeared pale. The size of infarction was determined by the following equations: Weight of infarction = (A1 x W1) + (A2 x W2) + (A3 + W3) + (A4 x W4) + (A5 x W5) + (A6 x W6), where A is percent area of infarction by planimetry and W is the weight of each section. Percentage of infarcted left ventricle = (weight of infarction/weight of LV) x 100.

### Cell culture and cell treatments

Primary NMCMs (neonatal mouse cardiomyocytes) were isolated as following steps. Briefly, the hearts of 1- to 3-day-old mice were taken out after being euthanized by decapitation, their ventricles were finely minced and then digested by 0.1% collagenase. Cell suspensions were collected, centrifuged, resuspended in complete DMEM medium (10% FBS, 100 U/ml penicillin and 100 μg/ml streptomycin) and plated for 1.5 hours under standard culture conditions (37°C in 5% CO_2_ and 95% O_2_) to allow fibroblast attach to the bottom. Meanwhile, because NMCMs rarely attached, the cell suspension mainly containing NMCMs was collected and plated into another culture dishes. We confirmed cell purity by both cellular morphology (beating cells) and immunostaining. Monoclonal antibodies directed against α-actin were used to positively identify cardiac myocytes.^23^ After 24 hours, NMCMs were attached and resuspended in Hanks buffer containing 1 mM MgCl_2_, 1 mM CaCl_2_ and 2% (w/v) BSA.

### Western blotting (WB)

The cells were washed with PBS for 3 times, lysed on ice with lysis buffer containing protease and phosphatase inhibitors and then centrifuged at 12,000×g for 15 min at 4°C. The resulting cell lysates were run on 7.5% or 12.5% SDS-PAGE gels and then transferred to PVDF membranes. These membranes were blocked with 5% non-fat dry milk in TBST for 1.5 hours at room temperature and then incubated with the appropriate primary antibodies overnight at 4°C. The membranes were subsequently washed with TBST for 3 tomes and incubated with a horseradish peroxidase (HRP)-conjugated secondary antibody for 1 hour at room temperature, after which proteins were visualized and quantified using a chemiluminescence western blotting detection system (Tanon, Shanghai, China). The protein expression levels of the target genes were quantified by relative densitometry and normalized to bands corresponding to β-actin, which was used as an internal control. The protein expression levels were presented as fold changes in relative to expression levels in the control sample. The experiment was repeated for 3 times.

### Measurement of cAMP in cardiomyocytes

We measured the cAMP concentration in NMCMs according the protocol of cAMP assay kits (Abcam, USA). Briefly, collected cells were washed with PBS for 3 times and then were added proper lysis buffer for 10 mins. After the 96 wells conjugated with HRP-cAMP, the test samples were added to these wells for 10 mins in temperature. Then wells were washed for 4 times with 1X washing solution. Finally, add 100 µL/well of Green Probe into each well, incubate at room temperature for 60 minutes to 3 hours and monitor the absorbance (OD value) increase at 405nm using an absorbance plate reader. The OD value of sample = OD^Positive^ - OD^Measure^ . OD^Positive^ refers to OD value of positive sample and OD^Measure^ refers to OD value of measurement of each sample.

### Ca^2+^ measurement in cardiomyocytes

Ca^2+^-loaded NMCMs were incubated with Fluo-3/AM (3uM) at 37°C for 45 min. For calcium transient assay, Ca^2+^-loaded The NMCMs were stimulated with carbamylcholine when imaged with the confocal microscope. Full duration at half maximum (FDHM) was used to measure the time duration of calcium transient.

### Fluorescence resonance energy transfer (FRET) assay

The NMCMs were transfected with TN-XXL pcDNA3 (Addgene, USA) for two days before experiments.^24^ Ca^2+^-loaded NMCMs transfected with TN-XXL were stimulated with carbamylcholine when imaged with the confocal microscope using excitation was at 430 nm. The peak fluorescence intensity of 475nm and 530nm were measured.

### Dual-luciferase reporter assay

To construct a luciferase reporter vector, we synthesized fragments of the ADCY6 3' UTR circ-HIPK3 and their mutant sequences, then inserted these fragments into a pmirGLO. For the reporter assay, we co-transfected NMCMs that were seeded in 24-well plates with either luciferase reporter construct and miR-17-3p mimic or negative control miRNA. At 48 hours after transfection, the cells were lysed, and luciferase activity levels were measured using a dual-luciferase reporter assay system. Firefly luciferase activity levels were normalized to Renilla luciferase activity levels. The experiment was repeated 3 times.

### Statistical analysis

Data was recorded and analyzed with SPSS 22.0 (IBM, Chicago, IL, USA). All data is presented as means ± SD or numbers and further analyzed by paired t tests. A P value of < 0.05 was considered statistically significant.

## Results

### The identification and effects of circ-HIPK3 in heart

**Figure 1:**
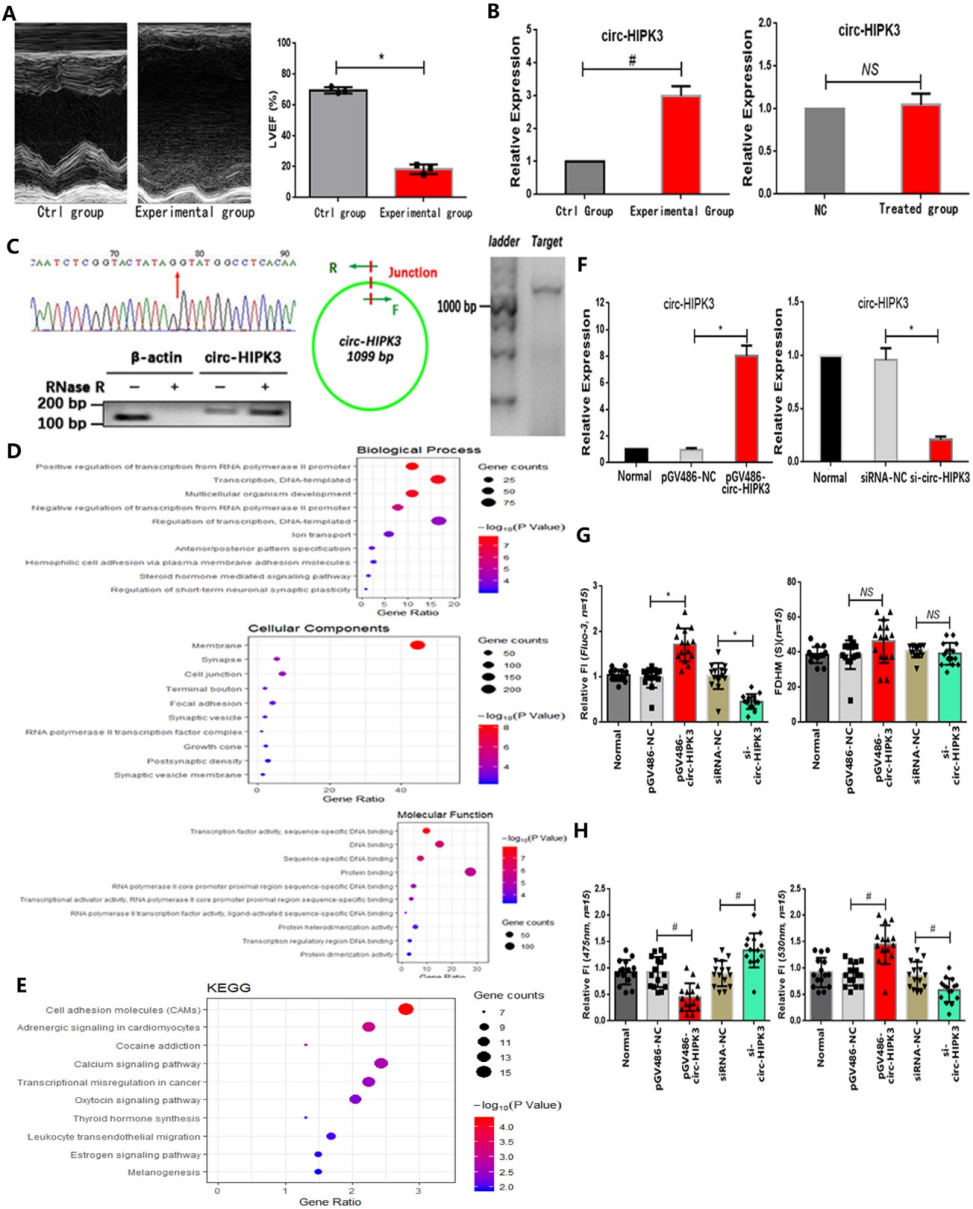
** The identification of circ-HIPK3. (A)** Left: Image of echocardiography showing enlargement of cardiac chambers and thinning of cardiac wall in experimental group; Right: Index LVEF decreased significantly in experimental group. * p < 0.05. **(B)** Two bar graphs showing that circ-HIPK3 increased significantly in experimental group but not in oxygen-glucose deprived CMs.* # p < 0.05, NS = not significant.* **(C)** Left upper: The result of Sanger sequencing of junction of circ-HIPK3. Red arrow points the junction; Left lower: Representative image of circ-HIPK3 digested by RNase R or not normalized by linear mRNA of β-actin; Middle: A pair of primer both containing junction can amplify the entire circ-HIPK3; Right: Agarose gel electrophoresis showed that the length of products was ~1099 bp. **(D)(E)** Bubble graphs showed the results of GO and KEGG analysis. **(F)** Left: The plasmids: pGV486-circ-HIPK3 can increase the level of circ-HIPK3 significantly; Right: the siRNA of circ-HIPK3 junction can decrease it remarkably. * p < 0.01. **(G)** Left: Results showing that circ-HIPK3 overexpression can increase the peak fluorescence intensity (FI) of Fluo-3 and vice versa; Right: bar graphs showing that up- or down-regulation of circ-HIPK3 had no influences on the time duration of calcium transient (FDHM). * p < 0.05, NS =not significant, n = 15. **(H)** Left: the circ-HIPK3 overexpression can decrease the FI of 475nm while its reduction can increase the FI; Right: the 530nm FI was increased by circ-HIPK3 overexpression and decreased by reduction. *# p < 0.05, n = 15.*

In our work, we found that both circ-HIPK3 and HIPK3 increased remarkably in hearts from HF mice post MI evidenced by echocardiography in relative to those in control group.(Figure 1A) Then we detected them in 12h oxygen-glucose deprived NMCMs but no variation was found, suggesting that circ-HIPK3 might be not associated with energy metabolism in heart.(Figure 1B) Circ-HIPK3 derived from the second exon of HIPK3 and its back-splicing junction fit GT-AG pattern of a U2 intron as previous researches.^7^ The positive results of Sanger sequencing of circ-HIPK3 junction, RNase R toleration and successful amplification of ~1099 bps products demonstrated the existence of circ-HIPK3 in heart.(Figure 1C) Above results showed that circ-HIPK3 indeed existed in cardiomyocytes (CMs) but the factor affecting its expression in HF were still unknown.

To explore the possible relationship of circ-HIPK3 with heart, we predicted its downstream targets by bioinformatic analysis on the basis of its canonical functional pattern of serving as a sponge for miRNAs. GO analysis of these targets showed that the most enriched BPs were positive regulation of transcription from RNA polymerase II promoter (GO: 0045944), most enriched MFs were transcription factor activity, and most related CCs were RNA polymerase II transcription factor complex (GO:0090575). KEGG analysis showed the most enriched pathways were Cell adhesion molecules (CAMs) (hsa04514), Adrenergic signaling in cardiomyocytes (hsa04261) and Calcium signaling pathway (hsa04020).(Figure 1D/E) Above results showed that circ-HIPK3 might be related to adrenergic signaling or calcium pathway at transcription level, so we further explored whether it affected the Ca^2+^ distribution (the key co-mediator of these two pathways) in CMs. We up- or down-regulated the circ-HIPK3 with pGV486-circ-HIPK3 and si-circ-HIPK3 (small interfering RNA) respectively. In order to verify the successful formation of circ-HIPK3 by plasmids, we transfected them into HEK293 cell line that didn't contain circ-HIPK3 and the positive results of Sanger sequencing of circ-HIPK3 junction from HEK293 cell line showed that circ-HIPK3 was generated without exotic nucleic acids being inserted.(data not shown). We found that the overexpression of circ-HIPK3 can increase the Ca^2+^ concentration in cytoplasm, while the under-expression had an opposite effect evidenced by Fluo-3 and FRET (fluorescence resonance energy transfer) assays. But the variation of circ-HIPK3 had no effects on the time duration of calcium transient.(Figure 1F-H) Above all, circ-HIPK3 can affect Ca^2+^ distribution in CMs but the underlying mechanism needed to be elucidated.

### The miR-17-3p mediates effects of circ-HIPK3 in NMCMs

**Figure 2:**
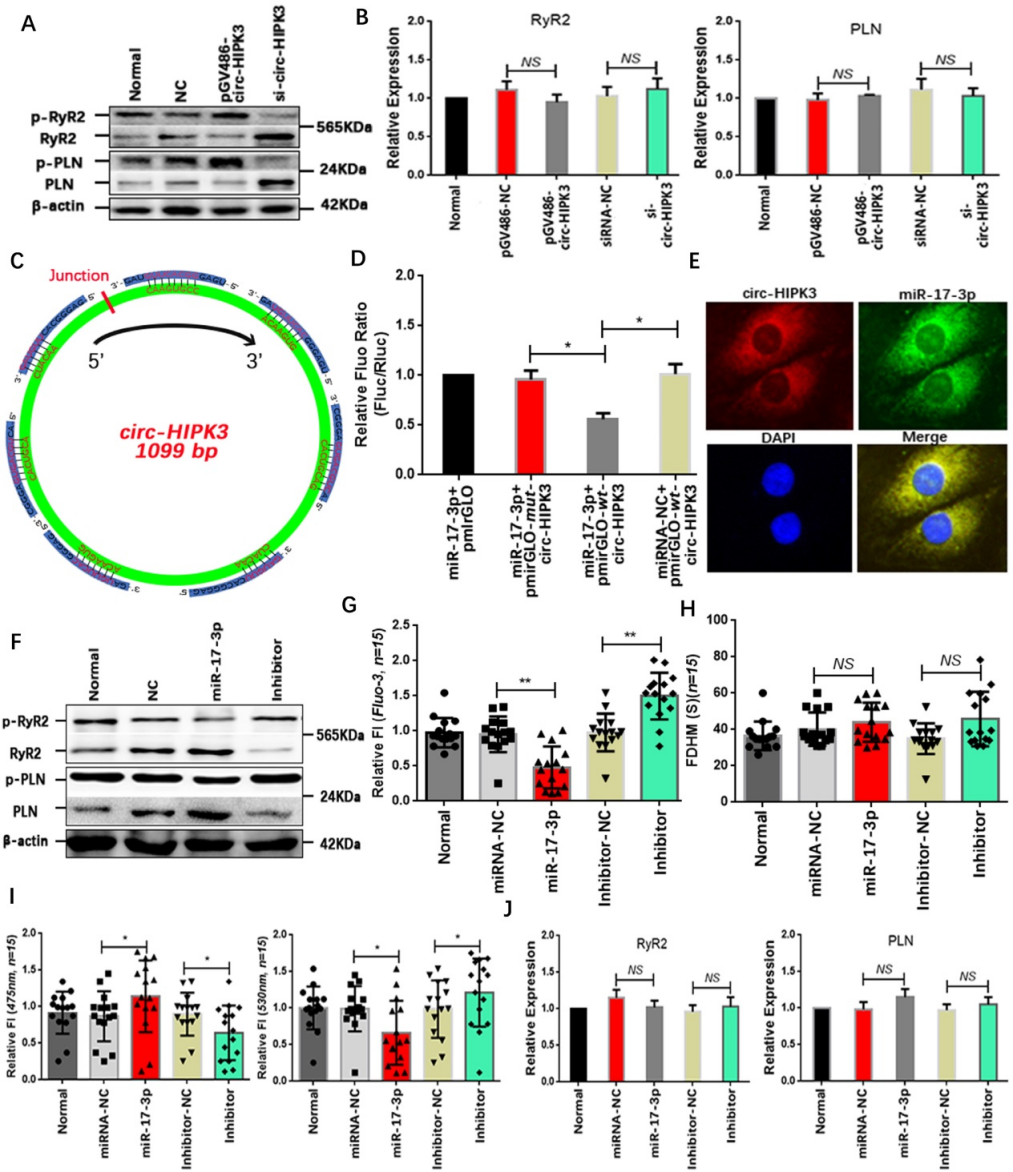
** MiR-17-3p was the downstream target of circ-HIPK3. (A)** Results of WB showing that p-RyR2 and p-PLN increased in circ-HIPK3 overexpressed NMCMs and vice versa. **(B)** QRT-PCR showing that PLN and RyR2 changed little in circ-HIPK3 over- or under- expressed NMCMs.* NS = not significant.* **(C)** Schematic image showing that seven possible binding sites of miR-17-3p to circ-HIPK3 were found. **(D)** Dual-luciferase reporter gene assay showed miR-17-3p could decrease the fluorescence density of NMCMs transfected with pmirGLO-*wt*-circ-HIPK3. * p<0.05. **(E)** FISH assay showed circ-HIPK3 (red) can interact with miR-17-3p (green), which mainly were around the nucleus (blue). **(F)** The level of p-RyR2 and p-PLN were downregulated by miR-17-3p mimic or upregulated by inhibitor. **(G)** The peak FI of fluo-3 decreased in miR-17-3p transfected NMCMs and increased in inhibitor transfected NMCMs. ** p < 0.01, n = 15. **(H)** The FDHM of NMCMs activated by carbamylcholine was not affected by miR-17-3p or inhibitor. *NS = not significant, n = 15.* **(I)** The miR-17-3p can decrease the FI of 530nm and increase the 475nm in NMCMs; Inhibitor can increase the FI of 530 and decrease the 475nm. * p < 0.05, n = 15. **(J)** Both miR-17-3p and inhibitor had no effects on PLN and RyR2 in genetic level. *NS = not significant.*

Above results showed that circ-HIPK3 overexpression can increase the peak concentration of Ca^2+^ in cytoplasm but not the time duration, implying that both SERCA2a and RyR2 were activated. The WB results that p-RyR2 and p-PLN increased significantly in circ-HIPK3 overexpressed NMCMs and vice versa supported this hypothesis.(Figure 2A) But no change of RyR2 and SERCA2a at genetic level suggested the variation of p-RyR2 and p-PLN was mainly associated with the activation of phosphorylase.(Figure 2B) To explore the mechanisms underlying the above phenomenon, we sought the downstream target of circ-HIPK3 by bioinformatics analysis and found miR-17-3p might be an ideal candidate because of circ-HIPK3 containing seven possible binding sites.(Figure 2C) However, whether circ-HIPK3 directly interacted with miR-17-3p was still a puzzle. Wide type (*wt*) and mutant (*mut*) circ-HIPK3 were cloned into a pmirGLO dual-luciferase expression vector and then were co-transfected into NMCMs with miR-17-3p mimic or negative controls. Normalized luciferase activity levels were significantly lower in cell co-transfected with miR-17-3p mimic and pmirGLO-wt-circ-HIPK3 than that in control groups.(Figure 2D) And FISH (fluorescence *in situ* hybridization) was further employed to show the interaction between circ-HIPK3 (red) and miR-17-3p (green) by probes labeled with PE (Phycoerythrin) or FITC (Fluorescein isothiocyanate).(Figure 2E) Above results demonstrated that miR-17-3p could interact with circ-HIPK3 directly. We next explored whether miR-17-3p affected the Ca^2+^ distribution in NMCMs. We pre-transfected NMCMs with miR-17-3p mimic or inhibitors for 24 hours, and peak concentration of Ca^2+^ in cytoplasm decreased significantly following the reduction of p-RyR2 and p-PLN in NMCMs transfected with mimic, and opposite effects can be observed in inhibitor-transfected NMCMs. But there were still no remarkable variation in time duration of Ca^2+^ transient and the level of RyR2 and PLN at genetic level.(Figure 2F-J) The similar level of circ-HIPK3 and miR-17-3p (*3:1*) further supported the hypothesis that circ-HIPK3 can influence the Ca^2+^ distribution in NMCMs via miR-17-3p. The above results suggested that miR-17-3p might be a bridge between circ-HIPK3 and calcium in heart.

### The ADCY6 is the target of circ-HIPK3 in NMCMs

**Figure 3:**
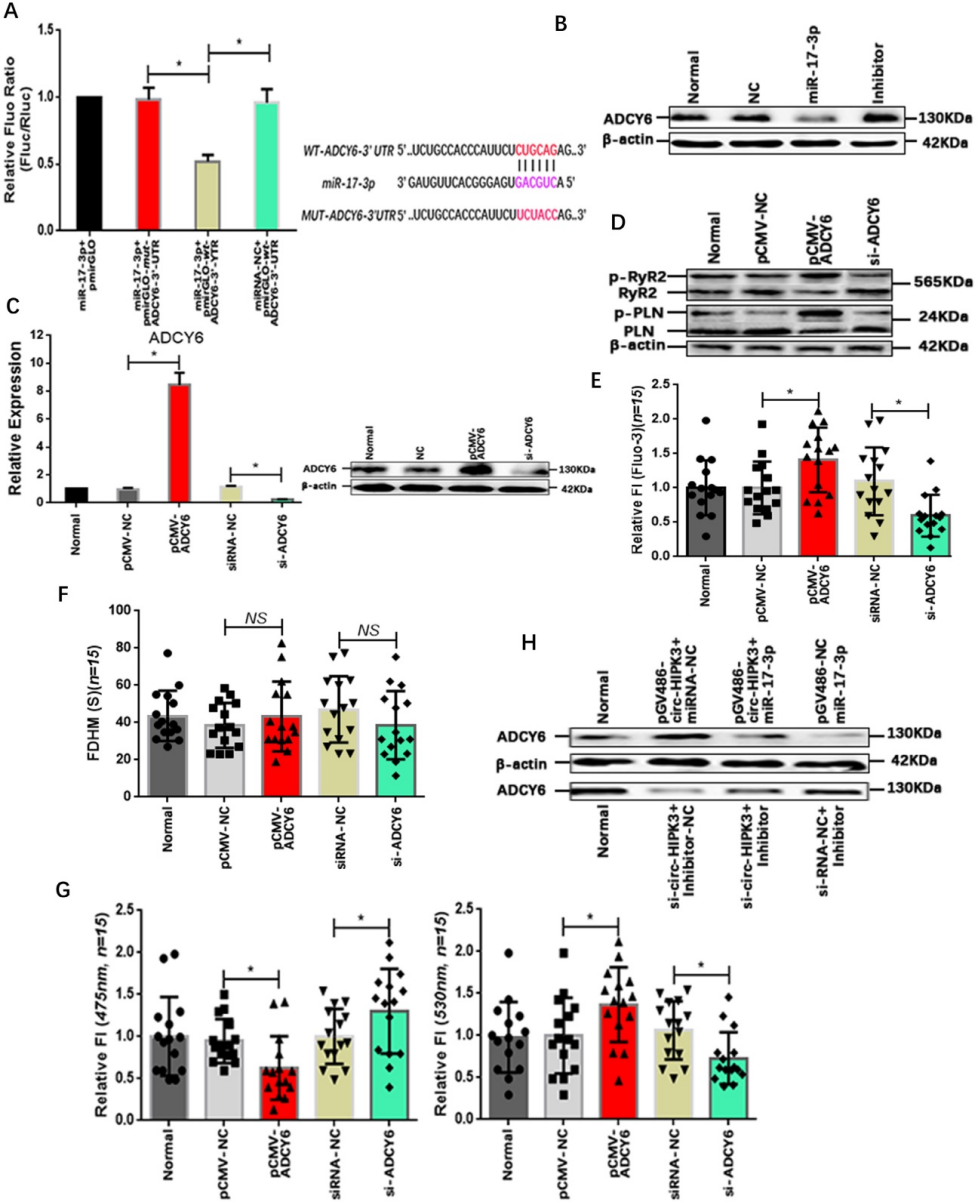
** Verification of existence of circ-HIPK3-miR-17-3p-ADCY6 axis. (A)** Left: Bar graph showing that miR-17-3p could significantly decrease the fluorescence density of NMCMs with pmirGLO-wt-ADCY6-3'UTR; Right: The binding sites of *WT* and *MUT* sequence in ADCY6 3'UTR with miR-17-3p. * p < 0.05. **(B)** WB showing that miR-17-3p can decrease the level of ACDY6 and inhibitor can increase it. **(C)** ADCY6 can be up- or down-regulated by pCMV-ADCY6 or si-ADCY6 at genetic and protein level. * p < 0.05. **(D)** The overexpression of ADCY6 can increase the level of p-RyR2 and p-PLN and vice versa. **(E)** The ADCY6 overexpression can increase the peak FI of fluo-3 in NMCMs activated by carbamylcholine and vice versa. * p < 0.05, n = 15. **(F)** Bar graph showing the variation of ADCY6 in NMCMs had little effects on FDHM of Ca^2+^ transient. *NS = not significant, n = 15.* **(G)** Left: The peak FI of 475nm can be downregulated by ADCY6 overexpression and upregulated by its under-expression; Right: The peak FI of 530nm can be upregulated by ADCY6 overexpression and downregulated by its under-expression. * p < 0.05, n = 15. **(H)** WB showing the increase of ADCY6 caused by circ-HIPK3 can be attenuated by miR-17-3p and the reduction induced by si-circ-HIPK3 can be rescued by inhibitor.

MiRNA, a key member of regulation network in cell, mainly exert its function by suppressing the translation via binding to the 3' UTR (untranslated region) of mRNAs. Bioinformatic analysis showed that the 3'UTR of ADCY6 (Adenylate cyclase type 6) contained one potential target site for miR-17-3p. ADCY6 functions in signaling cascades downstream of beta-adrenergic receptors in the heart and was closely related to the Ca^2+^ handling.^25^ To confirm the interaction of miR-17-3p with ADCY6, we again cloned the 3' UTR of ADCY6 or mutant sequences into pmirGLO vector that was co-transfected into NMCMs with miR-17-3p mimic or negative controls. Normalized luciferase activity levels were significantly lower in cells co-transfected with miR-17-3p mimic and pmirGLO-wt-ADCY6-3' UTR than that in cells with negative controls.(Figure 3A) In addition, the level of ADCY6 decreased significantly in miR-17-3p transfected NMCMs and vice versa.(Figure 3B) Above results showed that miR-17-3p could affect the ADCY6 by interacting with the 3' UTR. To confirm the effects of ADCY6 on Ca^2+^ distribution in CMs, we up- or down-regulated the ADCY6 with pCMV-ADCY6 or si-ADCY6 respectively.(Figure 3C) Results showed ADCY6 overexpression increased protein p-RyR2, p-PLN and the peak concentration of Ca^2+^ in NMCMs, whereas a reduction in ADCY6 could decrease p-RyR2, p-PLN and Ca^2+^ peak. And the variation of ADCY6 also had little effects on the time duration of Ca^2+^ transient.(Figure 3D-G) But whether circ-HIPK3 affected ADCY6 via miR-17-3p still remained unknown. Protein ADCY6 was upregulated in NMCMs transfected with pGV486-circ-HIPK3, and this increase can be attenuated by miR-17-3p mimic. Conversely, the reduction of ADCY6 caused by si-circ-HIPK3 also could be rescued by Inhibitor.(Figure 3H) Above results suggested that circ-HIPK3 could affect the Ca^2+^ concentration in cytoplasm through circ-HIPK3 - miR-17-3p - ADCY6 axis.

### Adrenaline upregulate the expression of circ-HIPK3 by CREB1

**Figure 4:**
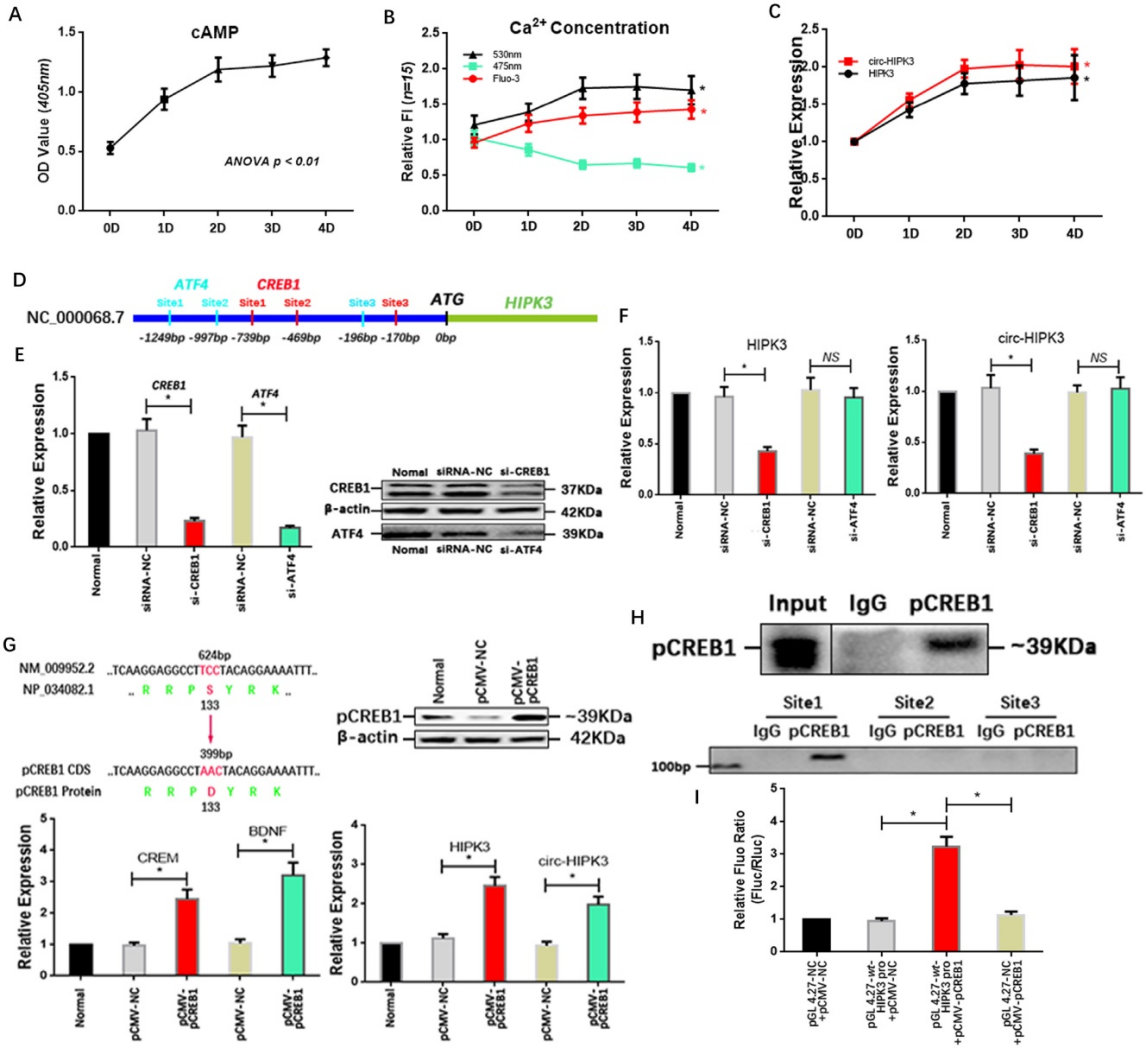
** Adrenaline increased the level of circ-HIPK3 by CREB1. (A)** The line chart showing that the cAMP increased significantly in adrenaline treated NMCMs with time and reached its almost peak at 2 day. **(B)** The FI of 530nm and Fluo-3 increased and 475nm decreased in adrenaline treated NMCMs with time and maintained stable after 2 day. * ANOVA, p < 0.05. **(C)** The level of HIPK3 and circ-HIPK3 increased with the treatment of adrenaline and reached their peak at 2 day which was consistent with the cAMP. * ANOVA, p < 0.05. **(D)** The schematic showing the promoter of HIPK3 containing three possible binding sites of ATF4 (green) or CREB1 (red) respectively. **(E)** The siRNA for CREB1 and ATF4 can decrease the expression of CREB1 and ATF4 at genetic and protein level. * p < 0.05. **(F)** Bar graphs showing that the reduction in CREB1 can decrease the level of HIPK3 and circ-HIPK3 but ATF4 can't. * p < 0.01, NS = not significant. **(G)** Upper Left: The schematic showing the 133 amino acid (serine) of CREB1 was changed into aspartate to mimic the serine phosphorylation; Upper Right: WB showing that pCREB1 was upregulated by pCMV-pCREB1; Lower Left: The known targets of CREB1: CREM and EDNF were upregulated by pCREB1 at genetic level; Lower Right: The level of HIPK3 and circ-HIPK3 increased in pCMV-pCREB1 transfected NMCMs. * p < 0.05. **(H)** Upper: The pCREB1 can be pulled down by anti-pCREB1 but not IgG; Lower: The sequences in site 1 can be pulled down by the pCREB1. **(I)** Bar graph showing that the increase of pCREB1 can upregulate the fluorescence density of reporter in NMCMs compared with negative controls. * p < 0.05.

Above results showed that circ-HIPK3 wasn't associated with energy metabolism but influenced the effects of adrenaline on calcium via ADCY6. So, we explored whether adrenaline affected the expression of circ-HIPK3 in CMs. In order to mimic the continuous stimulation of adrenaline in CMs, we cultured NMCMs with low dosage adrenaline (5umol/L) at consecutive time points (1d (day), 2d, 3d, 4d). Combined with the increase of Ca^2+^ in cytoplasm, the upregulation of cAMP (cyclic AMP) with time indicated cell model was built up successfully.(Figure 4A/B) Expectedly, the level of circ-HIPK3 and HIPK3 increased with time following the treatment of adrenaline and reached their peak concentrations at 2 days.(Figure 4C) But the underlying mechanism was puzzled. By analyzing the sequences (2000 bps) before HIPK3 in genome and adrenaline-related TFs (transcription factors) via JASPAR databases, we found that the possible binding sites of CREB1 (cAMP responsive element-binding protein 1) and ATF4 (activating transcription factor 4) can be identified.(Figure 4D) And qRT-PCR showed that HIPK3 and circ-HIPK3 decreased significantly in adrenaline-treated NMCMs transfected with si-CREB1 but not si-ATF4.(Figure 4E/F) To verify the above result, we increased the level of pCREB1 with pCMV-pCREB1 (*133-S to D*).

We first detected the level of its two well-known targets: CREM (cAMP-responsive element modulator) and BDNF (Brain-derived neurotrophic factor) in pCMV-pCREB1-transfected NMCMs to explore whether pCREB1 could exert its functions. The increase of them demonstrated that pCREB1 can work in NMCMs, and the following upregulation of HIPK3 and circ-HIPK3 supported the notion that CREB1 was associated with the variation of circ-HIPK3.(Figure 4G) To confirm the direct interaction between CREB1 and promoter of HIPK3, CHIP (chromatin immunoprecipitation assay) and luciferase reporter assays were employed. The PCR products of DNA pulled down by anti-pCREB1 showed that the first predicted binding site can interact with pCREB1.(Figure 4H) The *wt* or *mut* site1 of HIPK3 promoter were cloned into pGL 4.27 vector, and luciferase activity levels in cell co-transfected with pCMV-pCREB1 and pGL 4.27-*wt*-HIPK3 promoter were significantly higher than that in cell with negative controls.(Figure 4I) We further analyzed the franking sequences (500 bps) of circ-HIPK3 and found their relatively high reverse complementary rate (35%), implying that the increase in HIPK3 transcription could induce the upregulation of circ-HIPK3 via base pairing. The constant ratio (~2%) of circ-HIPK3 to HIPK3 further confirmed the conclusion. Above results showed that adrenaline can upregulate the expression of circ-HIPK3 via CREB1.

### Circ-HIPK3 overexpression *in vivo* improves cardiac function post-MI

**Figure 5:**
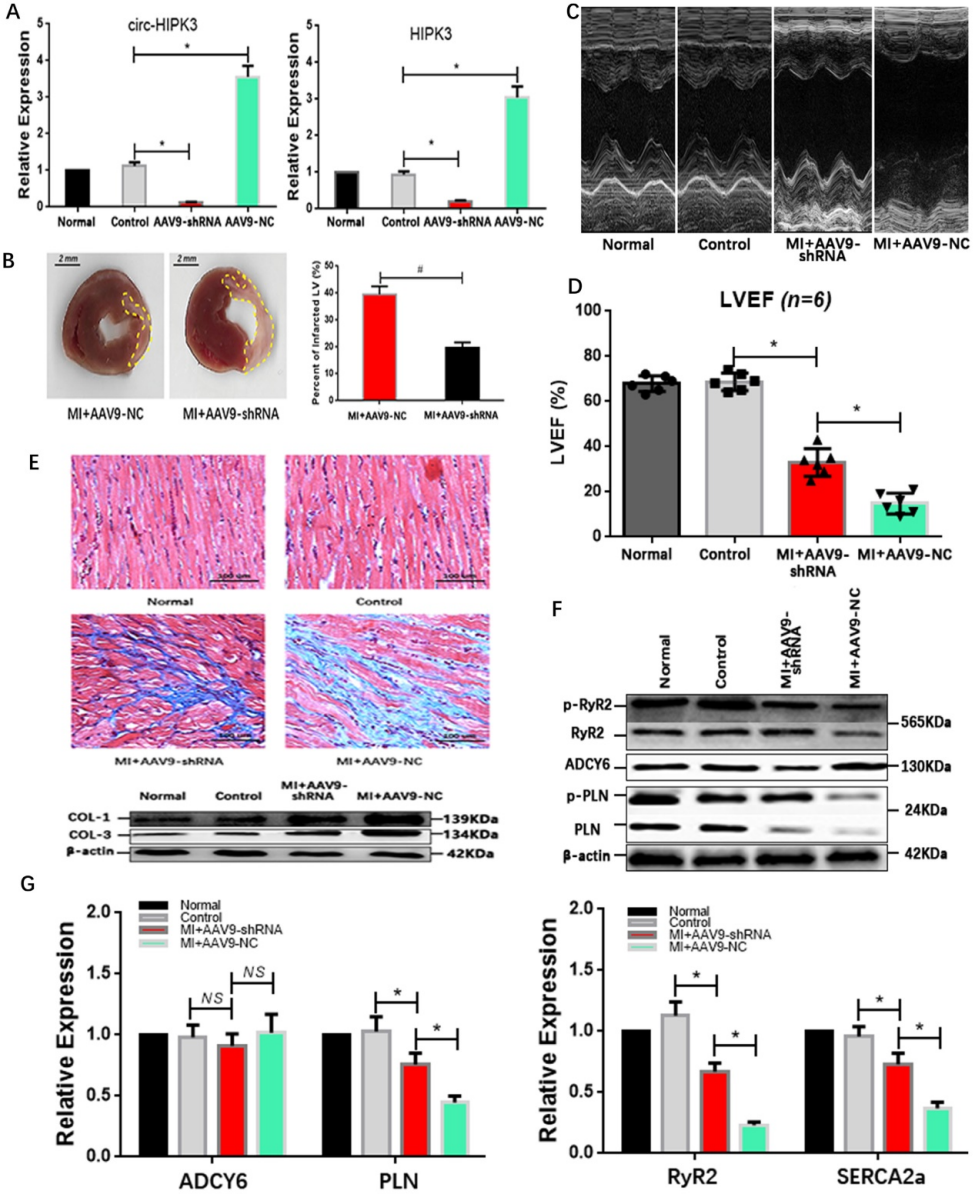
** AAV9-shRNA *in vivo* improved the cardiac function post MI. (A)** qRT-PCR showed HIPK3 and circ-HIPK3 increased in NC group and decreased in experiment group respectively. * p<0.05. **(B)** Left: Representative images of hearts sections by TTC staining (Viable myocardium stained red, and the infarcted areas appeared pale). Dotted box showing the infarcted areas. Right: Bar graph showing percent of infarcted LV in experiment group decreased significantly compared to that in NC group. * p<0.001. **(C)(D)** Results of echocardiography showed that the cardiac function of heart in experiment group increased significantly compared with that in NC group. * p<0.05. **(E)** Upper: Masson staining showed the degree of fibrosis of heart in NC group was much higher than that in experiment group, normal group or control group. Lower: WB showed col1 (collagen 1) and col3 (collagen 3) increased significantly in NC group. **(F)** Immuno-blotting showing that ADCY6 increased in NC group but the level of RyR2, PLN and their phosphorylated form decreased in NC group. **(G)** QRT-PCR showed that PLN, RyR2 and SERCA2a decreased significantly in NC group when compared to these in experiment group but ADCY6 changed little. * p<0.05, NS = not significant.

Our above results showed that circ-HIPK3 could be a helper of adrenaline in cardiomyocytes via upregulating ADCY6 *in vitro*. What the effects of circ-HIPK3 on heart *in vivo* was still elusive. Mice model of HF post MI were divided into four groups: Normal group (without surgery), control group (without ligation), experiment group (MI + AAV9-shRNA) and NC (negative control) group (MI + AAV9-NC). QRT-PCR showed the expression of HIPK3 and circ-HIPK3 were remarkably upregulated in NC group but downregulated significantly in experiment group.(Figure 5A)

Results of echocardiography showed hearts in experiment group had a better cardiac function in relative to that in NC group, and they also had a reduced infarct area evidenced by TTC staining (Triphenyltetrazolium Chloride).(Figure 5B-D) Consistently, the degree of heart fibrosis in NC group was much higher than that in experiment group.(Figure 5E) The above results suggested that AAV9-shRNA targeting circ-HIPK3 could partly alleviate the heart remodeling post MI and further maintain the cardiac function* in vivo*. Protein ADCY6 increased in experiment group as expected, p-RyR2 and p-PLN were relatively higher than those in NC group.(Figure 5F) And PLN, SERCA2a and RyR2 decreased significantly in NC group at genetic level, implicating that reduction in circ-HIPK3 could maintain the level of them.^26^(Figure 5G) Together, downregulation of circ-HIPK3 *in vivo* can alleviate the heart fibrosis and loss of cardiac function by maintaining the ability of Ca^2+^ handling in cardiomyocytes.

## Discussion

In our work, we identified circ-HIPK3 increased remarkably in heart post MI *in vivo*, and can affect the Ca^2+^ distribution in cytoplasm via miR-17-3p - ADCY6 axis. The circ-HIPK3 overexpression can increase Ca^2+^ concentration in cytoplasm and protein ADCY6, and this effect was ameliorated by miR-17-3p mimic. Protein ADCY6, as the direct target of miR-17-3p, could influence the Ca^2+^ distribution by PKA (protein kinase A). We also found that adrenaline could increase the expression of circ-HIPK3 via RECB1. And results *in vivo* showed that the reduction in circ-HIPK3 could improve the cardiac function post MI. Together, we found that the increase of circ-HIPK3 was beneficial to heart in the short term but harmful in the long run, and the reduction of it can maintain the cardiac function post MI *in vivo*.

The activation of sympathetic nervous system (SNS) responding to cardiac dysfunction could only exert a temporary support to heart. The longtime compensation for cardiac function needs humoral regulation such as the activation of β-AR by adrenaline. But this chronic activation harms the heart by disturbing loop of Ca^2+^ via dissociation of RyR2 subunit calstabin2 or reducing activity of SERCA2a in the long run.^27^, ^28^ As the key mediator of β-AR pathway, the accumulation of Ca^2+^ in cytoplasm can accelerate the hearts remodeling either by induction of atrial fibrillation (AF) or by activation of calcium-related pathways.^4^, ^29^ The special antagonist of β1-AR, β-blockers could reduce the mortality of HF significantly by maintaining the contractility and cardiac output or decreasing the possible of AF in HF, suggesting the importance of Ca^2+^ in the HF development.^30^ In our work, we found that circ-HIPK3 overexpression increased the concentration of Ca^2+^ in cytoplasm by phosphorylating RyR2 and PLN, whereas its reduction had an opposite effect. Experiments *in vivo* further demonstrated that a reduction in circ-HIPK3 could maintain the cardiac function by attenuating the remodeling of hearts via maintaining the expression of PLN, RyR2 and SERCA2a.^31^ The decreased level of them in NC group might be a negative feedback to the longtime overactivation of β-AR. But the exact mechanism of β-AR activation affecting the expression of PLN, RyR2 and SERCA2a need further studies.^26^ Together, the central role of circ-HIPK3 in heart was to enhance the effects of β-AR but impair the cardiac function in the long run.^19^-^21^

**Figure 6:**
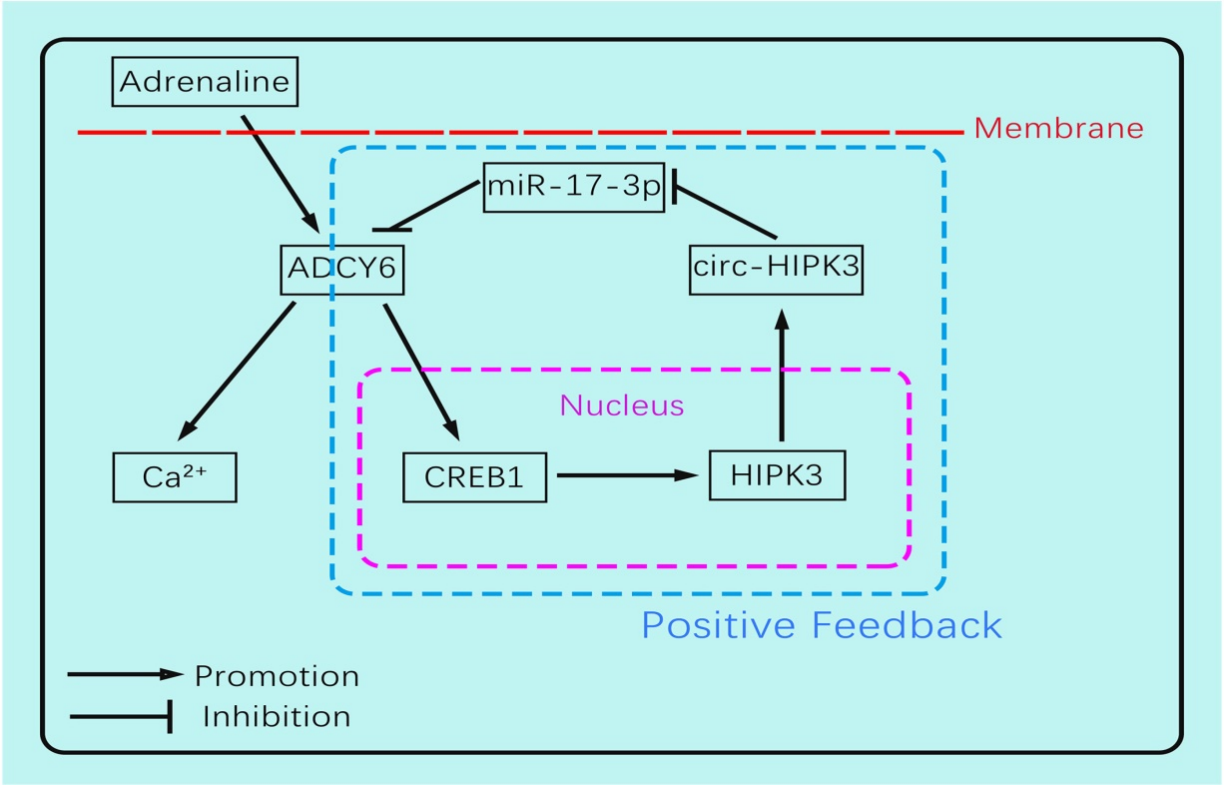
The schematic showing the mechanism by which circ-HIPK3 affects the Ca^2+^ distribution by miR-17-3p - ADCY6 axis and is regulated by adrenaline via CREB1.

The circ-HIPK3 generation was affected by adrenaline but not hypoxia or glucose-deprivation treatment, and enhanced the impacts of adrenaline to CMs by ADCY6. Our work showed that adrenaline can upregulate the transcription of pre(precursor)-HIPK3 via CREB1. Considering the fact that the formation mechanism of circ-HIPK3 was base pairing of franking introns, the increase of pre-HIPK3 could automatically upregulate the circ-HIPK3. So, there was a positive feedback between circ-HIPK3 and adrenaline, which can strengthen adrenaline's effects.(Figure 6) Meanwhile, the structurally stable of circRNA prolonged its effects duration to CMs and finally impaired the recycle of Ca^2+^. The downregulation of circ-HIPK3 *in vivo* can break the positive loop and also inhibited the effects of adrenaline to CMs via ADCY6. As the co-responder of β1- and β2-AR, the reduction of ADCY6 induced by circ-HIPK3 under-expression can exert a better inhibition for Ca^2+^ disorder in cytoplasm than β-blocks specifically targeting β1-AR. Above all, circ-HIPK3 might be an ideal therapeutic target for HF.

MiRNA, a key member of regulation network in cell, can serve as a mediator of circRNA by suppressing the translation of mRNAs. A bunch of miRNAs were associated with adrenaline or Ca^2+^ in cytoplasm in heart, and one miRNA also was associated with diverse phenotypes.^32^-^34^ By analyzing the results with bioinformatic analysis and assays, we first unveiled that miR-17-3p targeted ADCY6 and was sponged by circ-HIPK3. Apart from affecting Ca^2+^ in cell, miR-17-3p also can promote proliferation of cardiomyocytes in rat or mice to compensate the cardiac function via targeting metallopeptidase inhibitor 3 (TIMP3) or regulators of the PTEN-AKT pathway.^35^, ^36^ So, this might be another explanation of circ-HIPK3 associated with the mortality of HF. ADCY6 was a crucial mediator of β-AR to heart by activating the canonical cAMP-PKA pathway. Previous works showed that ADCY6 was also related to the myocardial hypertrophy, blood pressure or hyperdynamic cardiovascular response, suggesting that it played a crucial role in cardiovascular diseases.^37^, ^38^ Taken together, both miR-17-3p and ADCY6 were linked with the cardiovascular illness but this was the first time showing that they affected the cardiac function via Ca^2+^.

In conclusion, our work found circ-HIPK3 was related to the distribution of Ca^2+^ in CMs and further exerted the positive functions in the initiation, development of HF. The circ-HIPK3 expression was upregulated by adrenaline and can be a helper of adrenaline to promote cardiac function by accelerating Ca^2+^ handling via ADCY6. But its positive feedback would initiate and then worsen cardiac function by interfering Ca^2+^ distribution in cytoplasm and SR through circ-HIPK3 - miR-17-3p - ADCY6 axis in the long run. And its reduction improved cardiac function post MI significantly and attenuated the fibrosis degree. So, circ-HIPK3 could be a promising therapeutic target of HF.
